# Parameter-dependent stress states and their effects on properties of press-coated tablets

**DOI:** 10.1016/j.ijpx.2026.100610

**Published:** 2026-07-12

**Authors:** Henry Brauns, Chi Ki Leung, Jasper N. Ward-Berry, J. Axel Zeitler, Jan Henrik Finke

**Affiliations:** a Technische Universität Braunschweig, Institute for Particle Technology (iPAT), Volkmaroder Str. 5, 38104 Braunschweig, Germany; b Technische Universität Braunschweig, Center of Pharmaceutical Engineering (PVZ), Franz-Liszt-Str. 35A, 38106 Braunschweig, Germany; c Department of Chemical Engineering and Biotechnology, University of Cambridge, Philippa Fawcett Drive, Cambridge CB3 0AS, United Kingdom

**Keywords:** Press coated tablets, Press coating, Compaction analysis, Process parameters, Stress distribution, Tablet properties, Compactibility

## Abstract

Press-coated tablets (PCTs) are a modern dosage form designed to circumvent the negative effects of liquid and heat exposure that may damage drug substances during common wet coating processes for tablets. However, the production process is complex and the mechanical properties of the formulations of the different compartments may lead to challenges regarding processability, distribution of properties, and mechanical stability of the PCTs. In this study, materials with markedly different deformation properties were combined to elucidate the stress distribution during press coating and to assess the effects of process parameters and core geometry on the final PCT properties. Thorough structural investigations of all PCT compartments and the in-die analyses of axial and radial stress were applied. The simplistic approach to unify effects on the core properties by relating the punch force only on the projection area of the core only applies to the recovered core diameter and fails for further parameters. It accordingly requires more consideration of the complex stress states. The radial stress analyses clarify the anisotropy within the compression process of PCTs. It explains the findings on the distributions of porosity across different coat compartments and on the compactibilities of the recovered core. Finally, the breaking pattern of PCTs were explained with structural findings and the deduction of acting residual stresses.

## Highlights

• Radial stress measurement serves for the evaluation of anisotropy of stress states during press coating.
• Non-destructive Terahertz measurements prove less failure-prone than destructive manual evaluation of compartments.
• Core properties influence the heterogeneous stress distribution in the coat.
• Anisotropic stress states cause remarkable compactibility profiles of cores recovered from press-coated tablets.
• Structural differences elucidate the characteristic breaking patterns of press-coated tablets.

## Graphical Abstract

**Figure:**
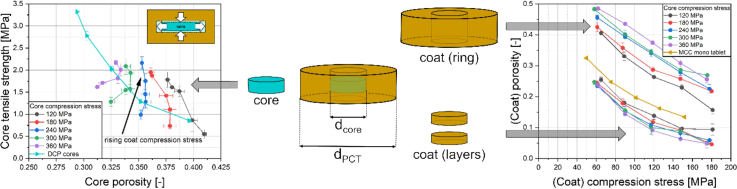

## 1 Introduction

The press-coating of tablets is an alternative to classical wet coating if the latter is inapplicable due to the material's susceptibility towards hydrolysis or heat exposure. In this approach, a drug-containing core tablet is compacted a second time inside a powder blend, which forms the coat (Kaljević et al., 2016). In addition to functional coatings (Lin and Kawashima, 2012), this process opens the possibility to combine different drugs or different release profiles in the coat and core compartments (Latha et al., 2011; Lin et al., 2004; Picart et al., 2022b). However, the production process of such press-coated tablets is complex (Rujivipat, 2010), as the state of the core before coating, as well as the process parameters during coat compression, interact to influence the final tablet properties. Based on its complex structure, different volumes of the final tablet must be differentiated, at least the core, the top and bottom layer of the coat and the outer annulus of the coat (Picart et al., 2021). In all these, the determining structural parameter, porosity, may be different compared to a mono tablet (Ascani et al., 2019; Diarra et al., 2015; Sinka, 2007) based on the deformation behaviour and process history of the components. Accordingly, application properties such as mechanical strength, disintegration, and dissolution may be altered.

The elastic recovery of different materials influences the tendency of the tablet to delaminate during and after the ejection (Mazel et al., 2013; Vaithiyalingam and Sayeed, 2010). A large difference in the elastic recovery of core and coating materials may lead to defects in the tablet structure. Cores with high elasticity cause delamination of PCTs made using a coat material with lower recovery. The increased expansion of the core causes stress that the core exerts on the coat. If this stress exceeds the bonding strength of the coat, cracks develop in the coat, eventually leading to delamination. However, even PCTs made using a combination of coating materials with high elastic recovery and core materials with low elastic recovery may lead to structural defects in the tablet. This is described by the effect of the expanding coat on the core. Cores with low strength exhibited cracking under this applied stress (Nguyen et al., 2020).

The measurement of the die wall stress and the related assessment of the axial-radial stress distribution have already been applied to compression processes of excipients, drug substances, and formulations (Abdel-Hamid and Betz, 2011; Michrafy et al., 2009; Obiorah, 1978; Windheuser et al., 1963). It was, inter alia, applied to identify the inverse correlation between crystal hardness and radial stress transmission (Higuchi et al., 1965) and to establish an easy method for assessing material plasticity without determining the material's true density, which is often challenging (Vreeman and Sun, 2022). The literature attributes different stress transfer patterns to materials exhibiting plastic deformation behaviour and to materials exhibiting brittle fracture behaviour during deformation (Carstensen and Toure, 1980; Cocolas and Lordi, 1993). For the analysis of press-coated tablets, Picart et al. applied the die stress analysis for the first time to elucidate the effect of the punch shape on the coating process (Picart et al., 2022c).

In this study, the effects of core and coating compression stress, as well as core diameter, on the structural properties of press-coated tablets and their separate compartments were investigated. Based on the differences in porosity across the compartments, the measured stress state and distribution during compression (axial and radial stresses), the mechanical strength, as well as the tablet fractioning pattern and the residual stresses within the final PCT, were deduced. In particular, our results concerning the porosity distribution and the formation of various fracture profiles are compared with the work of Picart et al. (Picart et al., 2022a; Picart et al., 2021) to extend their findings to new material combinations and deepen the analysis of axial-radial stress distribution.

The effects of anisotropic stress state during coat compression on core properties were investigated using radial strength measurements. The core was analysed after coat compression to assess geometric changes (height and diameter), resulting core porosities, and core tensile strengths. These changes in the DCP cores were compared with the results of changes in lactose and MCC cores from the work of Picart et al. (Picart et al., 2021).The altered physical core properties were compared with the results of other experiments involving complex compression (Carstensen et al., 1985; Meng et al., 2021; Pengjin et al., 2024) and decompression processes (Mazel et al., 2024; Radojevic et al., 2021).

To understand the influence of processing parameters in the complex manufacturing process of press-coated tablets (PCTs), the structure and mechanical properties were investigated in depth. The effects observed at the whole PCT level are traced to structural differences in the coat and core compartments, depending on process conditions and the geometry of the tablet core. To explain these, stress distributions and their effects are taken into account, and the overall effects on breakage patterns on the PCTs are elucidated.

## 2 Materials and methods

### 2.1 Materials

Microcrystalline Cellulose (MCC, Vivapur® 102) and dicalcium phosphate (DCP, Emcompress® anhydrous; both JRS Pharma, Rosenberg, Germany) were used as common excipients with drastically different particle deformation properties. Magnesium stearate (MgSt, Carl Roth, Karlsruhe, Germany) was used as a lubricant and colloidal silica (CS, Aerosil® 200, Evonik, Essen, Germany) was used as a flow aid.

### 2.2 Blending

MCC was blended with 0.25 wt.-% of CS, and DCP was blended with 2 wt.-% of MgSt with a Turbula mixer (Willy A. Bachofen, Switzerland) for 15 min at a frequency of 49 rpm, respectively. For readability, the abbreviations MCC and DCP will always refer to the respective blends with the low-concentration additive included. True densities of the blends were determined by Helium pycnometry (Ultrapyc 1200e, Quantachrome, USA) and used to calculate porosity.

### 2.3 Core compression

**Fig. 1:**
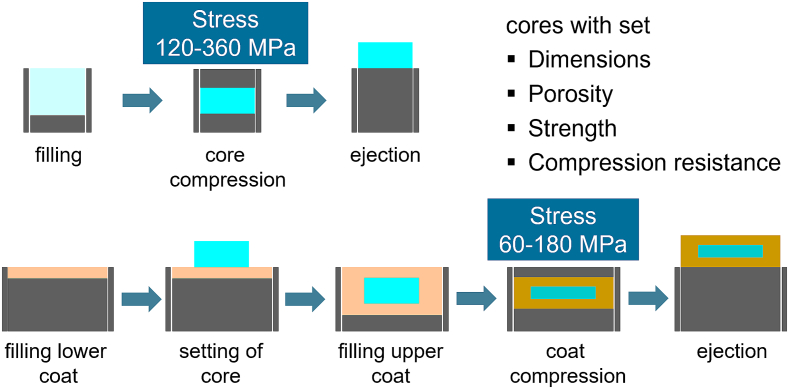
Schematic overview of press-coated tablet production (blue: DCP core; orange: MCC coating). (For interpretation of the references to colour in this figure legend, the reader is referred to the web version of this article.)

DCP cores were produced using a compaction simulator (Styl'One evolution, Medel'Pharm, Beynost, France) equipped with round, planar punches of the sizes 6.36 mm or 9 mm. DCP cores were compressed at 120 to 360 MPa (Fig. 1). The masses of the core tablets were adjusted for each core compression stress to possess the same final tablet height of 2.5 mm. All tablets were stored at lab conditions for 24 h before press-coating or testing.

### 2.4 Press coating

DCP cores were compressed with MCC as the coat compartment material. Using the same compaction simulator, approximately half of the coat powder was filled into an 11.28 mm die with planar punches, the core was placed on the powder bed centre manually and pressed in slightly, and the second half of the coat material was filled on top before compressing the formulation with 60–180 MPa (Fig. 1). Coat mass was adjusted to yield PCTs with a total mass of 500 mg. This approach was chosen to keep the outer geometry of the pre-compressed cores equal at the beginning of PCT compression while keeping the total PCT mass constant. Tablets were inspected after ejection, and defective PCTs were discarded before characterisation. As references, tablets containing only MCC or DCP, without cores, were produced with the respective weight and within the respective compression stress range. All tablets were stored at lab conditions for 24 h before testing.

### 2.5 Stress transmission

The transmission of applied axial compression stress to radial stress on the die wall was determined using an instrumented die for all 11.28 mm tablets. According to the manufacturer (Medel'Pharm, Beynost, France), the optimal position in the instrumented die (strain gauges) to achieve accurate results for radial stresses is a position of the mid of the tablet band at a depth of 6 mm in the process settings. This height was used for all tablets. The radial stress was plotted as a function of the axial compression stress to obtain the compression and decompression curves. As described by Vreeman and Sun, the slope of the compression curve in its terminal linear region corresponds to the stress transmission coefficient (STC). The addtool_quickfit:linear function in Origin 2025 was used for linear regression.

### 2.6 PCT characterisation

Produced PCTs were tested as a whole for weight, diameter, height and breaking force (MT50, Dr. Schleuniger, Switzerland). The MT50 measured at a constant moving jaw speed of 0.35 mm/s. The measurement was carried out in sensitivity mode with a force drop threshold of 50 mN. The measurement is stopped and the breaking force is determined from this first drop in force. The breakage patterns of the tablets were determined visually. During this process, fracture formation was continuously monitored during the breakage test to prevent multiple fractures from occurring. Tablets, for which the measurement continued beyond the first break, were rejected and not included in the analysis. Additionally, the ring of coating material (the band of the tablet, in contact with the die during compression) of untested PCTs was systematically removed with an abrasive metal hand file to expose the band of the core and the coat layers directly covering the top and bottom of the core. This sandwich was weighted and the coat layers were removed with a sharp blade to fully uncover the core. The core was again tested for weight, diameter, height, and breaking force. With this dataset, the masses, dimensions, and porosities of all compartments (core, coat layers, coat ring) as well as the mass, dimensions, porosity, and tensile strength of the whole PCT and the core were comprehensively assessable, respectively. For reference, all core tablets and 11.28 mm tablets only containing MCC or DCP were analysed for mass, dimensions, porosity, and breaking force. The tensile strength was in all cases calculated using the equation according to Fell and Newton (Fell and Newton, 1970):

(1) $$\sigma =\frac{2\cdot F}{d\cdot h\cdot \pi }$$

where *F* is the breaking force, *d* is the tablet diameter and *h* is the tablet height.

While the Fell and Newton equation is strictly derived for homogeneous, cylindrical, perfectly elastic bodies undergoing diametral failure, and therefore does not represent the true mechanical tensile strength of a complex PCT, we employ it here to calculate an apparent tensile strength. Because breaking force alone fails to account for the varying geometries of the PCTs produced in this study, scaling the load to failure by tablet dimensions provides a standardised, macroscopic index for comparative evaluation between PCTs and mono-material tablets.

The porosity of the whole tablet and the porosity of the compartments layers and ring were calculated in relation to the solid density $\rho_{s}$:

(2) $$\varepsilon =1-\frac{\rho_{\mathrm{tab}}}{\rho_{s}}$$

where $\rho_{\mathrm{tab}}$ is calculated from the ratio of mass to volume of the whole tablet (name overall porosity of PCTs) or the respective compartment of the tablet.

To quantify the diameter and height recovery, the radial and axial elastic recovery were calculated:

(3) $${\mathrm{ER}}_{\text{radial}}=\frac{d-d_{\min}}{d_{\min}}\cdot 100$$

(4) $${\mathrm{ER}}_{\text{axial}}=\frac{h-h_{\min}}{h_{\min}}\cdot 100$$

where *d* and *h* are the tablet diameter and height measured out of die and $d_{\min}$ and $h_{\min}$ the minimal diameter and height during the compression.

The max*imum theoretical stress acting on the core* (σ_core,max_) was calculated as a rough measure based on the assumption that the punch force can be applied directly to the projected core area:

(5) $$\sigma_{\text{core},\max}=\frac{4\cdot F_{\mathrm{ax}}}{{d_{\text{core}}}^{2}\cdot \pi }$$

where $F_{\mathrm{ax}}$ is the applied punch force and $d_{\mathrm{core}}$ is the core diameter.

### 2.7 PCT compartment porosity characterisation by terahertz time-domain spectroscopy

Selected intact PCTs and mono tablets were evaluated by non-destructive and non-invasive terahertz time-domain spectroscopy (THz-TDS) (TeraSmart, Menlo Systems GmbH, Germany). The characterised PCTs comprised core diameters of 9 mm and 6.36 mm, core compression stresses of 120 MPa and 360 MPa and coat compression stresses of 60 MPa and 180 MPa. For each PCT, a transflection measurement was conducted at the centre of each of the two flat tablet faces, and then a transmission measurement through the centre of the tablet in the axial direction. A total of three THz-TDS measurements were performed per PCT. Each measurement was obtained from averaging 50 acquisitions at 17 Hz in a nitrogen purged environment. The mass, thickness, and diameter of the PCTs were measured using a mass balance (PAS214, Fischer Scientific, France) and micrometer (Mitutoyo absolute 547–500 digital thickness gauge, Mitutoyo (UK) Ltd., UK), respectively.

Calibration sets of MCC and DCP tablets were respectively produced using a compaction simulator (HB50, Huxley Bertram Engineering Ltd., UK). MCC tablets were manufactured at five porosity levels between 0.1 and 0.3, with five tablets in each level. DCP tablets were similarly made with porosities between 0.37 and 0.47. All 50 tablets had a 10 mm diameter and weighed around 300 mg. THz-TDS transflection measurements were acquired for each tablet using the same configurations described earlier in this section. Linear calibration equations were derived for each material to relate the refractive index from transflection THz-TDS to the nominal porosity (Eq. (2)), and to relate the tablet thickness from THz-TDS to that from a micrometer (Mitutoyo absolute 547–500 digital thickness gauge, Mitutoyo (UK) Ltd., UK) to enhance accuracy. The micrometer was only used to measure the tablet thickness of the MCC and DCP homogeneous tablets, which were produced specifically for the THz-TDS calibration. The porosity, thickness and thus mass of the PCT compartments can therefore be evaluated.

## 3 Results and discussion

To account for the complexity of PCTs, different levels of observation and interpretation must be considered. For the further processing of PCTs, the relation between the overall properties of the dosage form and outward stresses is critical (Section 3.1). To describe the structure in detail, trace it back to process effects, and correlate it with the overall properties, deeper observation at the level of the individual compartments of the PCTs is needed (3.2, 3.3). In joint evaluation with the radial-axial stress data from the compression (Section 3.4), the reasons for the emergence of certain failure patterns in the PCTs can also be rationally explained (Section 3.5).

### 3.1 Influences on overall properties of PCTs

**Fig. 2:**
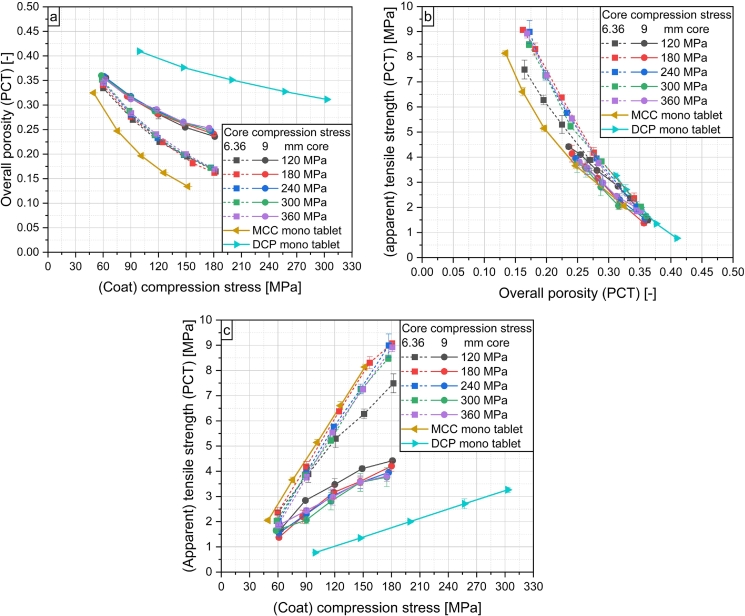
Apparent compressibility (a), compactibility (b) and tabletability (c) for PCTs; all compared with values for mono material tablets.

The overall PCT tablet properties lie between those of the materials in both compartments, as expected given the drastically different deformation behaviour: brittle in DCP and ductile in MCC. The apparent tensile strength of PCTs is determined by applying the Fell and Newton equation as a practical, apparent measure. This is because the assumption of homogeneous tablets and a single diametric breakage is not true for PCTs. However, this approach provides a reasonable measure for breakage resistance against an outer force impact related to the outer geometry. It therefore enables a reasonable and practical comparison between PCTs and homogeneous tablets without claiming the meaning of a purely physical value. Consequently, this section also discusses the apparent tabletability and apparent compactibility of PCTs. The apparent tabletability of the PCTs ranges between those of the separate materials. The apparent tabletability of PCTs with smaller cores is very close to the high tabletability of the MCC itself, while PCTs with larger cores range at lower values in the middle between MCC and DCP (Picart et al., 2021) (Fig. 2c).

By changing the core diameter between 6.36 and 9 mm, the mass relations between coat and core are also altered (Table 1 and Table 2). With smaller DCP cores, more MCC coat material is present (70–75 wt.-% for 6.36 mm cores, 42–50 wt.-% for 9 mm cores). Additionally, with the chosen approach of using identical core heights for all cores regardless of diameter and prior core compression stress, the mass ratio of the core to the whole PCT changes as well (25–30 wt.-% for 6.36 mm cores and 50–58 wt.-% for 9 mm cores from low to high core compression stresses, respectively). Furthermore, the approximate ring thickness of the coat is 2.46 mm for small and 1.14 mm for large cores. All these parameters lead to a clear advantage for PCTs with small cores, which possess a higher fraction of the high-binding MCC coat material and, geometrically, a higher cross-sectional area within the tablet that is filled with this highly compressible and compactible component. With the exception of 120 MPa core compression for 6.36 mm cores, all apparent tabletability values align with their MCC fraction (Fig. 2c). The selected core compression stress accordingly affects the apparent tabletability. The lower the core compression stress, the more pronounced the core deformation, which in turn influences the stress distribution and compression of the coat. High-porosity starting cores result in PCTs with higher breaking force and apparent tensile strength, in line with the literature (Ascani et al., 2019).

**Table 1:** Masses of the core and the entire PCTs.

Core diameter	Core compression stress [MPa]	Core mass [mg]	PCT mass [mg]				
			Coat compression stress [MPa]				
			60	90	120	150	180
6.36 mm	120	131.2	506.7	508.2	506,4	503.4	509.3
	180	138.5	502.7	502.1	501.8	501.6	496.4
	240	144.2	504.2	505.3	504.3	501.9	503.7
	300	150.5	497.0	495.7	495.5	496.9	496.7
	360	153.4	499.5	498.6	498.0	497.8	500.3
9 mm	120	258.6	514.7	513.0	511.6	509.5	507.5
	180	276.2	508.1	507.6	507.5	507.1	506.8
	240	284.4	506.3	504.8	504.7	504.7	505.5
	300	294.3	504.9	506.2	508.2	509.6	510.1
	360	300.3	510.6	509.6	509.6	510.6	510.7

**Table 2:** Mass ratio of the core to the whole PCT.

Core diameter	Core compression stress [MPa]	Mass ratio [%]				
		Coat compression stress [MPa]				
		60	90	120	150	180
6.36 mm	120	25.3	25.2	25.3	25.4	25.1
	180	26.7	26.7	26.7	26.7	27.0
	240	28.0	27.9	28.0	28.1	28.0
	300	29.6	29.7	29.7	29.6	29.6
	360	29.9	30.0	30.0	30.1	29.9
9 mm	120	49.7	49.9	50.0	50.2	50.4
	180	53.2	53.3	53.3	53.3	53.4
	240	55.4	55.5	55.6	55.6	55.5
	300	57.4	57.2	57.0	56.8	56.8
	360	58.0	58.1	58.1	58.0	58.0

The compressibility of the PCTs ranges between that of the two mono materials. At the same compression stress, MCC exhibits the lowest porosity and DCP the highest, corresponding to its highest and lowest compressibility, respectively. This difference increases with increasing compression stress (Fig. 2a). PCTs with smaller cores exhibit lower overall porosities, closer to those of pure MCC tablets, but still slightly higher than pure MCC. This is due to the higher compression-resistance of pre-compressed DCP core. For larger cores, the overall compressibility is obviously reduced, because, in the same way as for the apparent tabletability, the lower proportion of MCC in the coat combined with a higher proportion of DCP, leads to increased overall porosity.

Compactibility shows that all apparent compactibilities of PCTs are again between the curves for pure MCC and DCP. However, PCTs with smaller cores now better align with the compactibility of DCP, yielding about 40% higher apparent tensile strength (e.g., in the range of 0.22–0.25 porosity) than 9 mm core PCTs. These PCTs with larger cores closely match the pure MCC material and do not reach porosities below 0.23 at the highest investigated PCT compression stress of 180 MPa (Fig. 2b).

**Fig. 3:**
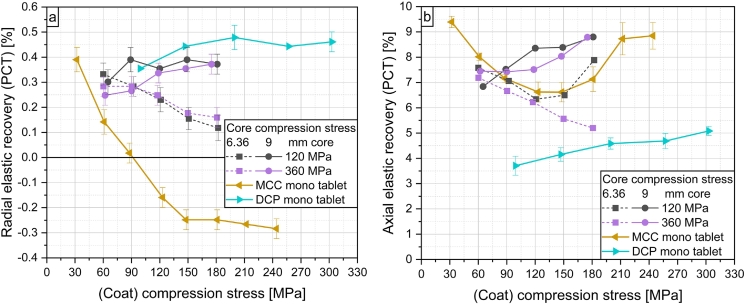
Radial (a) and axial (b) elastic recovery of PCTs compared with values for mono material tablets.

A key factor in the processability and quality of PCTs is the deformation behaviour of the materials and formulations across the different compartments. One often neglected, but very important parameter in PCT tabletting is the axial and radial expansion of the tablets after ejection. The final diameter and height of the single-compartment material tablets demonstrate their differences in this respect (Fig. 3a, b).

DCP shows a pronounced diameter expansion that rises with compression stress. On the other hand, MCC shows much lower expansion at very low compression stresses, even reversing to a constriction relative to the die diameter (11.28 mm), which increases with rising compression stress above 100 MPa (Fig. 3a). This is in line with the literature (Cabiscol et al., 2018; Picker, 2001; Zakowiecki et al., 2025). The different influence of the two materials is evident in the diametrical expansion of the PCTs. The interaction between a larger DCP core and a lower MCC volume fraction in the coat is particularly pronounced at high coat compression stresses. PCTs consistently show an increase in tablet diameter, despite the outer coat material being MCC. However, the core size influences the diametrical expansion: larger cores show an expansion (of the whole PCT) closer to the properties of pure DCP tablets, while smaller cores show the same trend of falling diameters with rising compression stress as MCC does. Nevertheless, they start at the same level of expansion as the larger cores and fall with a shallower (close to linear) slope than MCC, and accordingly do not reach the tablet-diameter constriction in the evaluated stress range (up to 180 MPa). The core compression stress has no pronounced effect on the radial expansion.

Regarding axial recovery of mono-compartment tablets, MCC generally exhibits higher values, which initially decrease up to a compression stress of 150 MPa and then increase again. In contrast, DCP shows a slight, constant increase in compression stress (Fig. 3b), consistent with the literature (Mazel et al., 2013; Picart et al., 2022a). All PCTs, regardless of core size or core compression stress, exhibit higher axial recovery than DCP. Larger cores even show higher axial recovery than pure MCC tablets in a certain compression stress range (100–180 MPa). With small cores and low core compression stress, an almost identical curve to that of pure MCC (decrease until 150 MPa, then increase) was found. In contrast, small cores with high core compression stress show a constant decrease in axial recovery. This effect of lower core compression stress indicates a stress distribution in the MCC coat that more closely resembles the axial recovery behaviour of pure MCC, suggesting a more homogeneous stress distribution throughout the coat compartment.

### 3.2 Influences on coat compartments

**Fig. 4:**
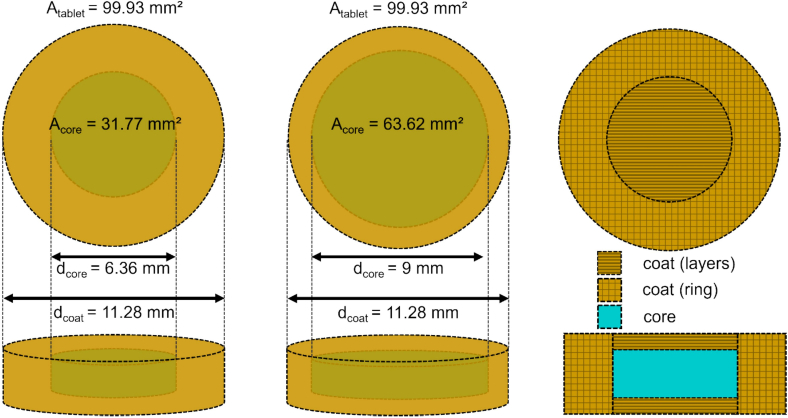
PCT, core dimensions, and compartment definition.

The changes in PCT characteristics hint at alterations to the properties of their compartments and that the geometric and process parameters mutually influence each other's effects on the PCT properties. The coat porosity is likely to influence the mechanical strength and other application properties of the PCT, such as the dissolution behaviour. The coat can reasonably be divided into two compartments based on the stress distribution (Fig. 4, right), as introduced in the literature (Picart et al., 2021; Sinka, 2007). One compartment is the coat layers on top of and below the core. Because the pre-compressed core has a higher resistance against deformation due to its already low porosity, the highest stresses are exerted on these coat layers (Picart et al., 2021). The second compartment, the coat ring, conversely experiences lower stresses because it consists only of loose coat powder in the direction of the major stress (axially).

It is accordingly obvious that the coat ring porosity remains higher than the porosity of the pure MCC tablet due to the limitation in compression from the resistance of the core. In contrast, coat layers experience higher compression stresses and are compressed to lower porosities (Fig. 6). This heterogeneous distribution of compressive stress during coat compression on the ring and the layers has already been observed by Picart et al., who simulated the density and stress distribution for MCC/lactose systems with the Finite Element Method (FEM) (Picart et al., 2022a; Picart et al., 2021). It depends on the core compression stress and on how much higher its resistance is at the centre of the PCT than in the coat layers, compared with that of the coat ring. At lower core compression stresses, the core exhibits lower resistance and deforms to a greater extent, more closely resembling the behaviour of the coat ring. Accordingly, the coat layer and ring porosity show fewer differences at low core compression stress. With rising core compression stress, differences between layers and rings become more pronounced, leading to a broader distribution of coat porosity. This may also contribute to the explanation of lower apparent tabletability due to higher core compression stresses (Fig. 2c), which convey higher compression resistances. A comparison of results for the two core diameters shows that smaller cores yield lower porosity in both coat compartments (Fig. 6). The lower porosity values suggest that the local stress in the layers and the ring is higher when smaller cores are used. These findings, based on the results of the experimental analysis in this study, are consistent with the findings from the FEM simulation data in the work by Picart et al., who performed such investigations for PCTs based on MCC and Lactose as main constituents (Picart et al., 2021).

**Fig. 5:**
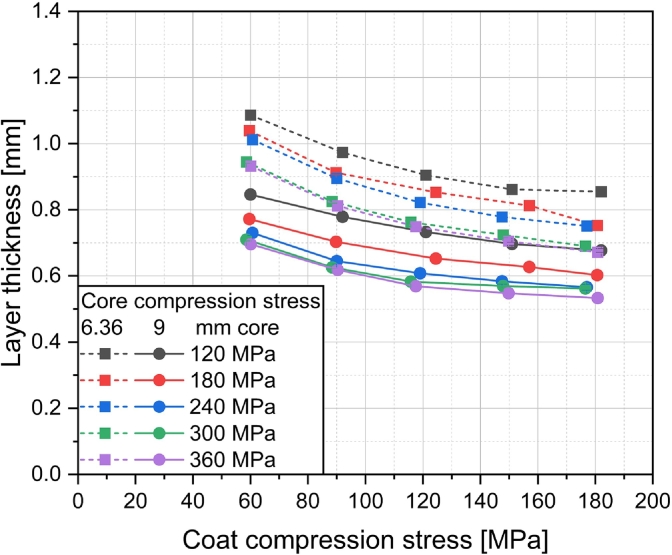
Layer thicknesses of PCTs.

The coat layer thickness was identified as a parameter influencing stress distribution in PCTs in previous work of Picart et al. (Picart et al., 2022a; Picart et al., 2021). Using FEM simulations, they demonstrated that the axial stress in PCT compartments with an 8 mm core and a 16 mm coating at an axial compression stress of 50 MPa varies with changes in layer thickness. While layer thickness is a critical variable influencing stress distribution (Picart et al., 2021), the experimental design of the present study inherently couples core mass with layer thickness to maintain a constant total PCT mass of 500 mg. As shown in Fig. 5, the layer thicknesses in this study vary by less than 0.2 mm across populations. Drawing on the numerical simulations by Picart et al., which demonstrated a 7.3% variation in maximum axial stress over a much larger 67% change in layer thickness, we conclude that while layer thickness undoubtedly contributes to the observed structural variations, the primary driver of the macroscopic effects discussed in the following sections remains the core compression stress.

**Fig. 6:**
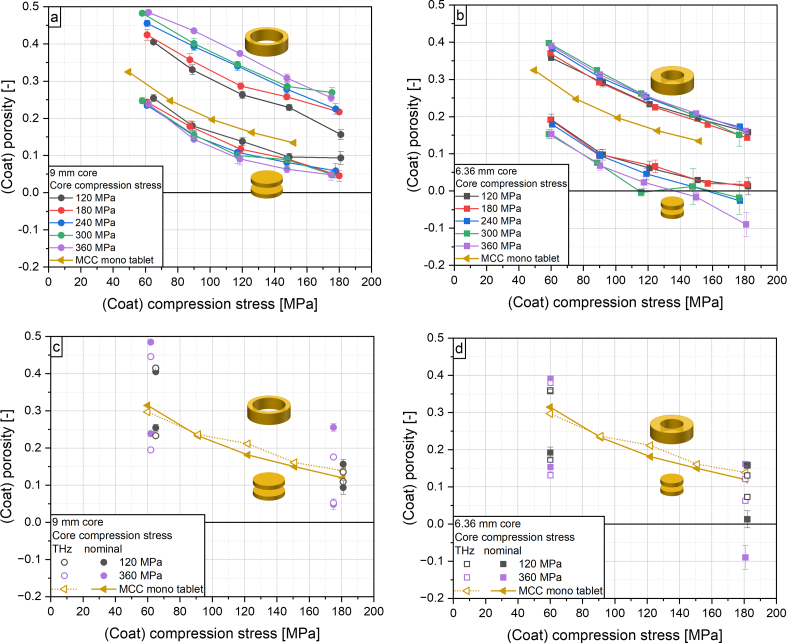
Influence of the core compression stress and the coat compression stress on the coat ring and coat layer porosities of PCTs with 9 mm (a) and 6.36 mm (b) cores. Comparison of porosities measured with THz (*n* = 1) and the nominal porosities of PCTs with 9 mm (c) and 6.36 mm (d) cores.

The negative out-of-die porosities for the coat layers are physically impossible (Fig. 6b). Reasons for the measured porosity falling below zero may include an excessively high coat layer mass value or an excessively low coat layer volume value. Both of these values are included in the coat layer porosity calculation. An excessively high coat layer mass value would result from not removing all of the mass belonging to the core or the coat ring during the preparation of the MKT. In this case, the coat layer mass would be calculated as too high, resulting in an underestimation of the true porosity. The coat layer volume is calculated using the height of the entire MKT and the core height after complete removal of the coat. It may be the case that the core axially relaxes after the complete removal of the coat. This recovery is considered in the calculation of the coat layer volume, resulting in an underestimation of the coat layer volume and porosity. Due to the error susceptibility of this method, the non-destructive method of THz-TDS measurement presents itself as a reasonable alternative for more direct measurement of densities and porosity.

A subset of the PCTs and a new series of MCC mono tablets were measured using THz-TDS to non-destructively and non-invasively determine the porosities of the coat ring and coat layer and to compare it with the porosity of the mono tablets (Fig. 6c, d). THz-TDS allows measurements of intact PCTs without material loss, while maintaining their geometric integrity. The porosities derived from THz-TDS are thus slightly different but more realistic than those from the destructive characterisation method, as no negative values are obtained for porosity (Fig. 6d). The THz-TDS results agree with and validate the deductions presented above. Further analysis of PCT compartment properties using THz-TDS and a detailed discussion of the methodology will be presented in a separate publication. Nonetheless, the initial results demonstrate the promising capability of THz-TDS in quantifying PCT compartment properties with greater convenience, efficiency, and effectiveness. The non-destructive nature of THz-TDS enables the same PCT to be evaluated for its stresses, elastic recovery, porosity, and apparent tensile strength. Such complete and systematic analyses of PCTs shall be explored in future.

### 3.3 Influences on core properties

**Fig. 7:**
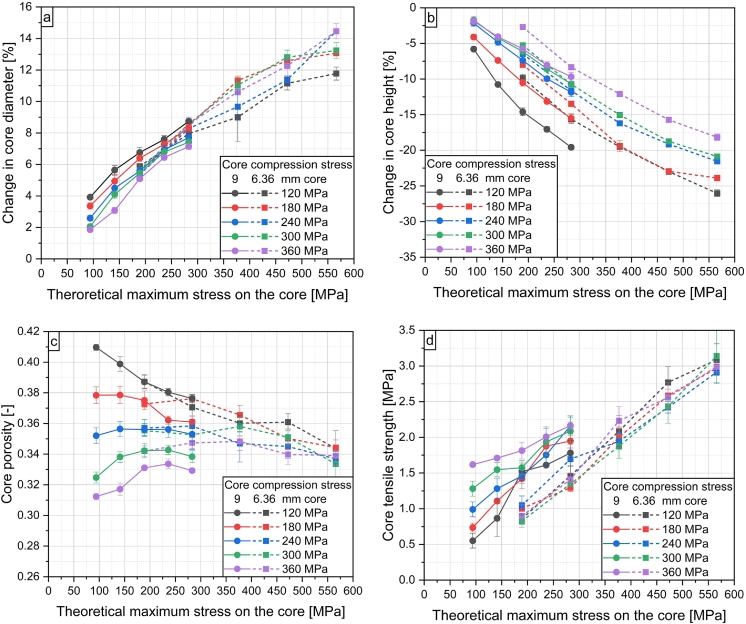
Correlation of the core diameter change (a) and core height change (b), core porosity (c), and core tensile strength (d) with the theoretical maximum compression stress on the core.

Based on the differences in porosity over the coat compartments (Fig. 6), a low core compression stress generally leads to greater homogeneity in the coating porosity. This is because the core still strongly deforms during coat compression, enabling a higher densification of the coat annulus by 1) the core's lateral expansion (Fig. 7a) and 2) its loss in height (Fig. 7b). The stress state during compression of the overall system, consisting of a pre-compacted tablet core in a loose powder bed, is initially influenced by the inhomogeneous resistance to deformation arising from the presence of the already strong core. Seen from PCT surfaces, which are in contact with the punches, the tablet consists of an outer ring that only contains loose coat powder and a centre that contains the pre-compacted core tablet and layers of coat powder on top and below (Fig. 4). Accordingly, the compression resistance is higher in the centre area because of the core's compact, more mechanically resistant form compared with only the loose coat powder. The size of this centre region is defined by the size of the core tablet. The distribution of axial stresses over the centre and ring of the punch contact area is accordingly correlated to the projection area of the core perpendicular to the (axial) stress direction (Fig. 4). As a first-order heuristic approximation, we evaluated whether the stress acting on the core could be scaled proportionally to the punch force divided by the core projection area (σ_core,max_, Eq. (5)). By testing this boundary condition, we aimed to determine if core properties could be unified across different core sizes without requiring numerical modelling. However, as demonstrated in, this rudimentary scaling factor ultimately fails to unify the structural properties across different geometries. This empirical limitation underscores the complexity of PCT mechanics, indicating that unrepresented variables, such as layer-thickness variations and triaxial stress gradients, preclude such simplifications and necessitate multi-variable evaluations for accurate property prediction. The stress acting directly axially on the coat ring must conversely be much lower than the global stress exerted by the punch surface, which is usually calculated as a compression process parameter by dividing the compression force by the punch projection area.

The lateral expansion of the core as a result of the axial stress transmits additional stresses to the coat ring due to its confinement between the core and the die wall. This stress and volume reduction, in turn, reduces the porosity of the coat ring under a complex, anisotropic stress state. The lateral expansion of the core during PCT compression is mainly a function of σ_core,max_ (Fig. 7a). Accordingly, this parameter appears to be a reasonable scaling factor as it unifies the curves for different core diameters. The core height is indeed continuously decreasing with increasing coat compression stress, but it shows a significant discrepancy across core sizes when plotted against σ_core,max_ (Fig. 7b). The values for porosity and tensile strength of the different core sizes, which are determined from the core height and diameter, cannot be unified using σ_core, max_ either (Fig. 7c, d). Uniformity of the core properties across different core sizes was therefore only observed for the change in core diameter. In particular, for more complex structural and property parameters, no uniformity could be achieved and it was not possible to identify a common range in which the values overlapped for different core sizes. This can be attributed to the highly simplified approach and, in particular, to the fact that the varying layer thicknesses (Fig. 5) may possess an additional impact in this analysis (Picart et al., 2021). It can therefore be concluded that this simple assumption σ_core, max_ is not suitable for reliably unifying core properties across different core sizes. Possible extensions to account for layer thickness and material characteristics could represent an approach for future developments of simplified models that need a low number of readily assessable parameters and that do not need FEM simulations.

Cores with a lower core compression stress continuously lose porosity over σ_core,max_, while cores with higher core compression stress initially gain porosity at low σ_core,max_ due to the initially high lateral expansion of the core. Above 250 MPa σ_core,max_, curves for all core compression stresses take a unified progression with a slightly negative slope. This hints at a common overall structural state of these cores caused by a more homogeneous stress state surrounding them. For smaller cores, this state is reached at lower coat compression stresses. This emphasises that the ratio of core and coat projection area has a direct influence on the stress states achievable under common process parameters for coat compression.

The concept of compactibility describes the direct dependence of tensile strength on porosity in a compact, assuming the material is identical. This holds for most die compression processes with a single homogeneous compartment. In the case of PCT cores (recovered from the PCTs), this is obviously not the case, as the core porosity and core tensile strength (Fig. 7c, d) follow a different trend. This accordingly suggests that the highly anisotropic stress states in the PCT compression are also causing structural differences compared to those resulting from die compression of homogeneous compartments.

**Fig. 8:**
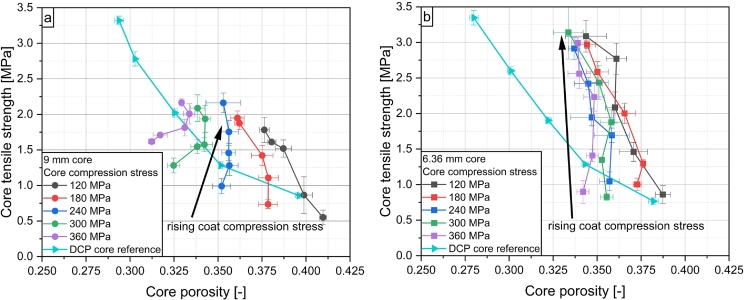
Influence of core and coat compression stress on the compactibility of 9 mm (a) and 6.36 mm (b) cores. References are the compactibilities of DCP in the respective core dimensions before coat compression.

Comparison of the compactibilities of core tablets before and after PCT compression (recovered from the PCTs) shows a generally different correlation between tensile strength and porosity, depending on the stress states experienced during PCT compression (Fig. 8). Such a different correlation between the evolving tensile strength and density of cores prepared from PCTs has also been analysed by Picart et al. for MCC and lactose cores in MCC coat (Picart et al., 2021). For larger cores with high core compression stress, the compactibility curves run most untypically from low to high porosity (with rising coat compression stress) while increasing the tensile strength, completely opposite to typical progression, such as for the reference cores (without press coating, Fig. 8a). This trend of increased strength in less dense cores was observed in the analysis by Picart et al. for smaller lactose cores. In contrast, no higher strength was achieved with larger lactose cores compared to the reference cores (Picart et al., 2021). For large cores with low core compression stress, the typical direction (lower porosities, higher tensile strength) prevails, but with a much steeper slope than for the reference cores. For small cores, compactibility is less dependent on core compression stress, in line with its lower effects on core porosity and tensile strength (Fig. 7c, d). Small changes in porosity lead to a considerable increase in the tensile strength of the cores, far beyond that of the reference cores. Because of that, the recovered cores from PCTs yield much higher tensile strength (up to 150% higher) than those produced in uniaxial die compression. This trend regarding the compatibility of the cores was observed by Picart et al., particularly in the case of small cores made from MCC (Picart et al., 2021).

These findings demonstrate the effects of the three-dimensional stress state and the compression history of the cores. Basic findings on such effects by varying core dimensions, but not for different core compression stresses in PCTs, were published by Picart et al. (Picart et al., 2021), while the fundamentals of strength anisotropy based on anisotropy of triaxial stress states in homogeneous materials were discussed by Galen and Zavaliangos (Galen and Zavaliangos, 2005). They showed for die compression that ductile powders exhibit higher strength in the transverse direction (common direction of tablet testing) than in the compressive direction. The opposite was shown for brittle powders. Overall, anisotropy is dependent on the compression pressure: higher pressures result in more isotropic behaviour. Particle shape, particle overlap, pore orientation, and contact mechanisms were identified as particularly important factors influencing the anisotropy, as key variables for controlling strength and its directional dependence.

Due to the more complex stress state in the cores during coat compression compared to die compression, comparisons with investigations of compression processes using a triaxial setup can be drawn. In general, the literature shows an increase in the strength of various materials under radial confining compression stresses (Meng et al., 2021; Pengjin et al., 2024). However, it should be noted that the applied stresses in these triaxial setups do not allow for a direct comparison with those during PCT coat compression in terms of their direction and extent. The results of Carstensen et al. show the influence of triaxial compression on already compacted tablets, such as in PCTs (Carstensen et al., 1985). The second compression was carried out in a bed of non-compactable material. The result was a tablet with reduced height and mostly unchanged diameter (slightly increased). The comparison of compactibilities of these core tablets before and after the second compression shows the same correlation between tensile strength and porosity, and therefore cannot be related to the results of this work (Fig. 8).

Following triaxial compression, the cores also undergo a degree of triaxial decompression, influenced by the deformation behaviour of the coat material. Several studies have investigated the effect of triaxial decompression on tablet properties (e.g. compactibility). Recent work by Mazel et al. shows that the value of the die wall stress during decompression and ejection did not affect the density and tensile strength of round and planar tablets (Mazel et al., 2024). No significant difference was observed between the results MCC of the triaxial instrumented die and the rigid die. These results contradict previously published data by Radojevic et al., which show that triaxial decompression significantly influences tensile strength (Radojevic et al., 2021). However, those results compared the tensile strength of cylindrical tablets with that of cuboid tablets. The differences were therefore due to the tablet shape and the method used to determine tensile strength (Mazel et al., 2024). It is reasonable to assume that the compactibilities of the cores in PCTs are not affected by triaxial decompression. Furthermore, cores in a mechanically stable PCT coat cannot fully recover and remain subject to an anisotropic stress state after ejection.

Future analysis of these influencing parameters will enable a more detailed understanding of the anisotropic stress state in PCTs.

### 3.4 Analysis of the stress distribution

**Fig. 9:**
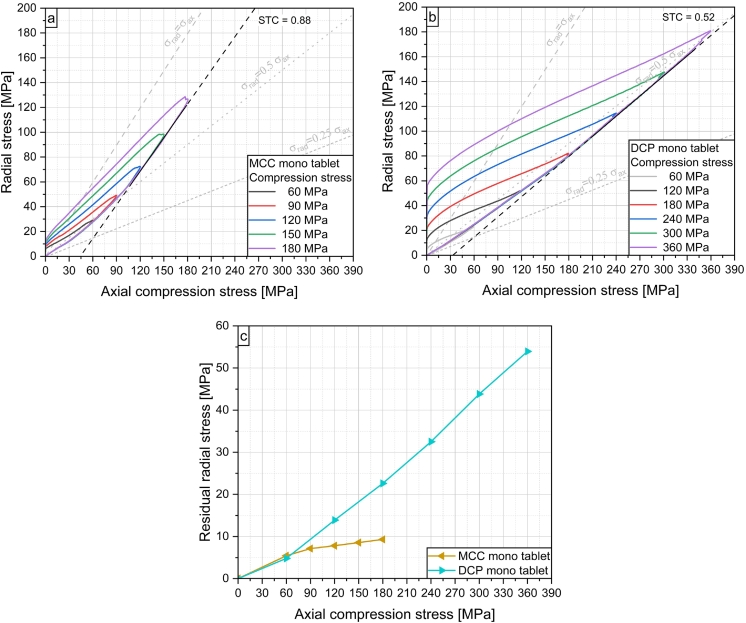
Relation of between axial and radial stress during mono tablet compression of MCC (a) and DCP (b); residual radial stress during mono tablet compression (c).

To prove the assumed effects of core properties on the properties of PCT compartments, die wall stress was analysed to gain information on the transmission of stresses from the axial to the radial direction. The stress transfer of the PCTs was compared to the stress transfer of the respective mono material tablets. The axial stress applied by the punches is increasingly radially distributed to the die wall with increasing compression stress for MCC (Fig. 9a). This initial bending of the curve is more pronounced for MCC than for DCP, while the latter also had a lower slope at high axial stresses (Fig. 9a, b). During decompression, DCP shows a flatter decline in radial stress than MCC as the axial stress decreases. DCP also shows higher values for radial stress than for axial stress at approximately 25–28% of the maximum axial stress. For MCC, this value is around 17–18% of the maximum axial stress.

**Fig. 10:**
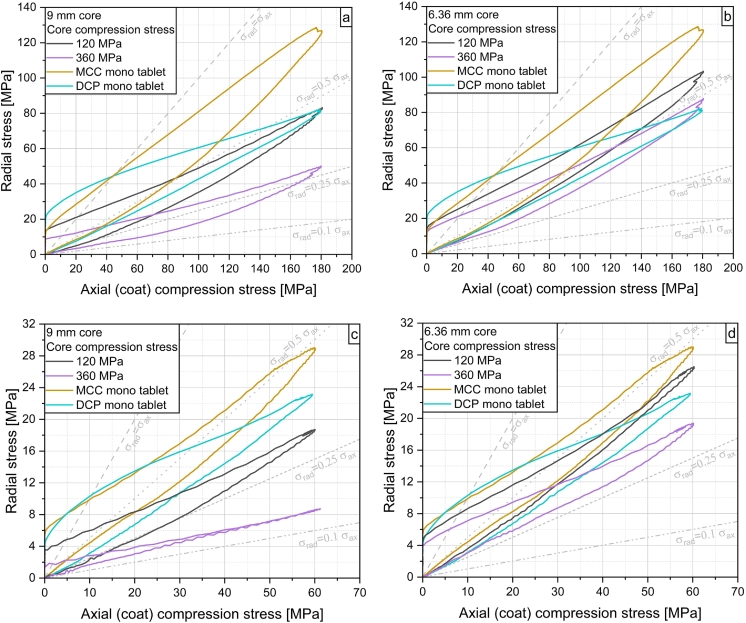
Influence of core compression stress on 9 mm (a, c) and 6.36 mm (b, d) cores on the relation of between axial and radial stress during PCT compression with 180 MPa (a, b) and 60 MPa (c,d). References represent MCC and DCP as homogeneous materials.

It is clear that the high transmission of radial stress to MCC is not achieved for MCC coats containing more resistant DCP core tablets (Fig. 10). At least in the low axial stress range, PCTs also show lower radial stresses than DCP. This again hints at the high axial stress concentration at the centre of the PCT over the core region. Due to the high deformation resistance of the core, the coat ring compartment is not strongly compressed because it does not experience high local, axial stresses either. Accordingly, lower stresses are transmitted to the die wall.

Towards higher axial stresses, the curves for PCTs bend more strongly towards higher slopes than DCP or MCC as pure materials. This may be indicative of the combination of material deformation properties in the compartments and the transition in their dominance, as more stress is transmitted to the die wall due to stronger core deformation. This provides two distinct stress components to the coat ring: (1) an axial compressive stress arises due to the yielding core height (Fig. 7b), and (2) an increasing radial stress due to the diametrical core expansion (Fig. 7a). That may finally cause the PCTs to show higher radial stresses (for specific axial stresses) than the DCP, but never higher than the MCC in the compression stress ranges investigated.

With higher core compression stress, the achieved radial stresses in PCT compression are lower. This is due to the higher core resistance to deformation resulting from its initial lower porosity. Accordingly, the transition to higher radial stresses would be expected to occur at higher axial stresses than for low core compression cores, if such a change occurs at all.

For smaller core diameters, higher radial stresses are reached, which are also more homogeneous and less dependent on the core compression stress. This confirms the expectations based on the findings that a higher σ_core,max_ in smaller core PCTs also caused more homogeneous coat (Fig. 6) and core porosities, core tensile strengths (Fig. 7c, d), and core compactibilities (Fig. 8). This further supports the theory that a more homogeneous stress distribution and also higher radial stresses may positively affect the overall apparent PCT compactibility (Fig. 2b).

With a higher coat compression stress of 180 MPa (Fig. 10a, b), the maximum radial stresses of the PCTs are closer to those of the reference materials (MCC and DCP) than at the low coat compression stress of 60 MPa (Fig. 10c, d). This suggests a more homogeneous stress distribution at higher coat compression stresses. This assumption can be confirmed by evaluating the porosity of the coat ring and coat layers using THz-TDS measurement (Fig. 6c, d).

Another difference between the two materials can be seen in the stress exerted on the die wall after the axial stress has completely subsided (Fig. 9c). This residual radial stress is influenced by the deformation behaviour (i.a. the elastic modulus) and the axial and radial elastic recovery of the respective materials. During die compression, the powder is deformed in such a way that die wall pressure is generated. Due to elastic recovery in the axial compression direction during decompression, this die wall pressure is reduced to a certain extent, again depending on the deformation behaviour of the material. The large relaxation in the axial direction of elastic materials results in low residual radial stresses. However, materials with plastic deformation behaviour deform with a high degree of irreversibility and form a large contact area with the die wall and cause in higher residual radial stresses as reported in the literature (Abdel-Hamid and Betz, 2011; Sugimori et al., 1990; Takeuchi et al., 2004). As the maximum axial stress increases, the residual radial stress of DCP mono tablets increases linearly, while that of MCC mono tablets levels off towards a fixed value (Fig. 9c), just as the radial elastic recovery tends towards a fixed value (Fig. 3a). The reason for this observation has also been discussed in the literature and explained by the strong bonding properties resulting from the high bonding strength in MCC, which is inter alia attributed to a high density of strong hydrogen bonds (Abdel-Hamid and Betz, 2011; Krycer et al., 1982).

**Fig. 11:**
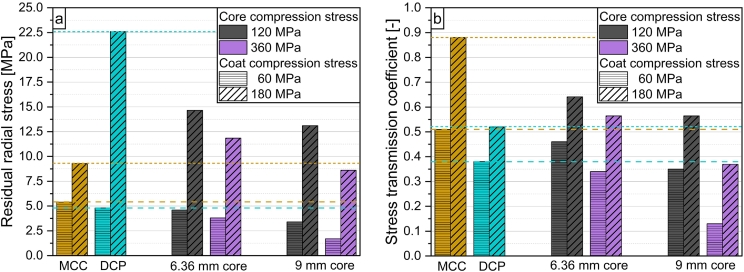
Effect of core diameter and core compression stress on residual radial stress (a) and stress transmission coefficient (b) in radial-axial stress analysis. Evaluation performed at maximum axial stress of 60 and 180 MPa.

The residual stresses on the die wall of PCTs (Fig. 11a) are mainly in the range between those of MCC and DCP. However, they are much closer to those of the outer coat compartment MCC for the coat compression stress of 180 MPa, which is in contrast to the PCT diameters, which are closer to those of DCP (Fig. 3a). An explanatory approach may be the combination of the soft, more elastic behaviour of the outer coat MCC in die and the timely expansion of DCP upon ejection. As expected, lower radial stress transmission also leads to lower residual radial stresses, as is true for stronger compacted cores. It is remarkable that PCTs with a 9 mm core at high core compression stress exhibit a residual stress even lower than that of MCC itself. This may indicate strong core resistance, leading to very low densification of the coat ring and high compression of the coat layers, resulting in a highly inhomogeneous density distribution (Fig. 6). The lower coat compression stress of 60 MPa shows a different result for the residual radial stress of the mono tablets: DCP tablets have a slightly lower residual stress on the die wall than MCC tablets (Fig. 9c). The lower the coat compression stresses, the closer the residual die wall stresses are to each other, which correlates with the same starting level of PCT diameters (Fig. 3a). The fact that the 9 mm cores exhibit the lowest residual radial stress at low coat compression stress, which is lower than that of MCC and DCP tablets, is consistent with previous findings. The most heterogeneous porosity distribution is expected in these PCTs. Larger cores, lower coat compression stresses, and higher core compression stresses show the most heterogeneous distribution.

The stress transmission coefficient (STC) was introduced by Vreeman and Sun (Vreeman and Sun, 2022) as a directly correlating measure of plasticity of mono materials, derivable from axial-radial stress measurements, without the need for careful calibration and determination of the true density. Its values for PCTs must again be regarded and marked as apparent values because no homogeneous specimen is tested, nonetheless providing a reasonable comparison on an applied level. Hence, these apparent STCs are again mostly between the STCs of DCP, with low plasticity, and MCC, with high plasticity. However, PCT values are, in this case, closer to those of DCP. This parameter indicates that the PCTs appear almost as non-plastic as DCP with respect to stress transition. It is clear that this measure cannot be interpreted as a direct measure of plasticity in the press-coating setting. Nevertheless, it provides an indication of the achievable general strength and the apparent overall plasticity of the PCT, which becomes noticeable during compression. The STC scales well with the residual radial stress, except for the pure materials (compare Fig. 11a, b).

### 3.5 Effects on breakage patterns of PCTs

**Fig. 12:**
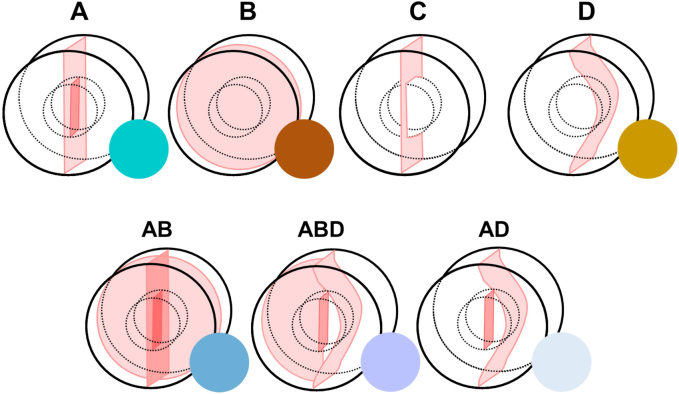
Breakage patterns of PCTs during diametral breakage testing. Extended classification based on the work of Picart et al. (A,B,C,D) (Picart et al., 2022a). Brown dots mark a breakage pattern without a breakage of the core, and blue dots mark a breakage of the core. (For interpretation of the references to colour in this figure legend, the reader is referred to the web version of this article.)

**Fig. 13:**
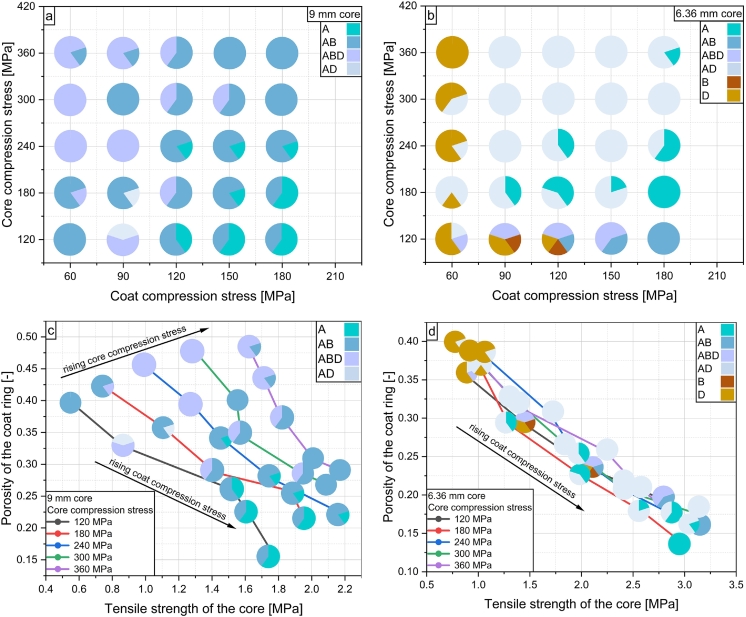
Breakage patterns depending on the process parameters of core compression and coat compression for 9 mm (a) and 6.36 mm (b) cores in PCTs; attribution of breakage patterns to coat ring porosity and core tensile strength as an effect of core and coat compression stresses for 9 mm (c) and 6.36 mm (d) cores in PCTs. Each dot represents a sample of *n* = 5.

The effect of structural differences in PCTs, caused by geometric and process variations, may influence their fractioning patterns during typical diametrical compression tests used to determine tablet breaking force. The breaking patterns introduced by Picart et al. (Picart et al., 2022a) were extended to include combinational patterns, including breakage of the core (A) with other fractioning patterns such as delamination (B) or breakage around the core (D), which occurred in the current study (Fig. 12). The resulting patterns are related to the process parameters of the core and coat compression stresses (Fig. 13a, b) and associated with the structural parameters of the porosity in the coat ring and the tensile strength of the core (Fig. 13c, d). Each circle in Fig. 13 consists of 5 parts representing the breaking pattern of a sample of *n* = 5 PCTs.

For large cores, leaving only about 1.14 mm ring thickness of the coat around the core, the core always breaks (A or combinations thereof), and the lateral ring at the height of the rim of the core appears to be a weak point (B or combinations thereof) (Fig. 13a). This weak point is additionally subjected to residual stress within the PCT. The core and coat consist of different materials with varying degrees of radial recovery (Fig. 2c). This difference causes residual stress between the core and coat in the radial dimension across the projected area of the larger cores. This residual stress must be contained by the bonding forces within the coat and promotes fracture failure at weak points in the PCT. At high coat ring porosities (induced by high core compression stresses and low coat compression stresses), a combination with breakage around the core prevails (ABD). This suggests low binding forces between the coat ring and core under low ring compression. The breakage pattern around the core indicates a greater difference in porosity between the coat layers and the ring, so that the break occurs along this boundary of differing porosity at the compartment boundary. This particular pattern of fracture has already been explained by Picart et al. (Picart et al., 2022a). Furthermore, the breakage around the core can be explained by residual stresses acting in the PCT after decompression and ejection. At low coat compression stress, the porosity in the layers is 0.23–0.25, and in the ring it is 0.4–0.48 (Fig. 6). Based on the compressibility of pure MCC (Fig. 2a), these porosities can be attributed to local stresses of 75–85 MPa in the layers and 20–30 MPa in the ring. These different local stresses in the two compartments result in differences in both radial and axial elastic recovery (Fig. 3a, b), leading to a residual stress difference at the interface between these two areas, which promotes failure along this boundary. As coat compression stress increases, delamination (AB) becomes more frequent. At the highest coat and lowest core compression stresses, total diametrical breakage (A) dominates, which most likely accounts for strong binding forces between the core and coat and a more homogeneous coat porosity distribution (Fig. 13c).

For small cores (Fig. 13b, d), coat breakage around the core is dominant (D) at low compression stresses, probably due to the thicker ring layer and its relatively lower strength (compared with the core). The breakage pattern (D) is also attributable to the residual stresses within the PCT coat and to the difference in porosity between the coat layers and the ring, as described in the previous paragraph. With higher core and coat compression stresses leading to denser coat rings and stronger cores, the combination of core breakage accompanied by breakage around the core (AD) is most frequent in contrast to larger cores. This suggests lower strength at the interface between the core and the coat, which is surprising given the high radial stresses measured (Fig. 10b) and the low radial elastic recovery (Fig. 3a). Conversely, very dense coat layers and large differences relative to less dense coat rings were analysed (Fig. 6b) and may lead to weak points in the structure at the contact points between the coat layer and the ring. Additionally, this is the shortest distance through the coat for such tablets compared to PCTs with larger cores, where the ring is the shortest distance through the coat. Accordingly, fewer delamination breakage patterns are observed for PCTs with small cores due to the high adhesion of the coat layers to the core.

Whether small or large cores, the interfacial adhesion between the individual compartments thus proves to be an important influencing parameter on the breaking force and fracture profiles of the entire PCT. They originate from differences in the structural distribution of porosity, which in turn are caused by the stress distribution arising from the material's deformation properties and the process parameterisation. Studies on the interfacial strength of bilayer tablets have identified a wide variety of factors that influence the layer adhesion. Similar elastic recovery of the materials (Busignies et al., 2013), higher surface roughness due to the use of coarse powder (i.e., large particle size) and low tamping stress of the first layer (Kim et al., 2023; Zhang et al., 2018), and convex punches (Zhang et al., 2018) were found to improve interactions between adjacent layers. The influence of the second layer compression stress in bilayer tablets has also been investigated in the past, and it has been shown that higher second layer stresses lead to higher interfacial bonding strength by generating a larger bonding area resulting from lower porosity. However, higher compressive stress in the second layer can also lead to a greater difference in radial elastic recovery between the layers and reduce the interfacial bonding strength (Chang and Sun, 2019). The transferability of these findings to PCTs and deeper studies of adhesion between the core and the coat should be examined in future research.

The evaluated fracture profile of a PCT can therefore provide an indicator of the strength of the bond between the core and the coat compartment layers and ring. The selected process parameters during coat compression influence the structural parameters of the PCT, which, together with the core properties, have a decisive influence on the breaking patterns.

## 4 Conclusion

The compression stress used to compact the core tablets for PCTs drastically affects their processability during press coating and the final PCTs' properties. Lower core compression stresses lead to a more homogeneous stress distribution in the axial and radial directions, yielding more similar porosities in the coat ring and the coat layers. A homogeneous distribution of these porosities may provide a mechanically more stable coat. Smaller core tablets transmit axial stress more strongly in the radial direction because the effective stress on the core is higher due to its smaller projection area, leading to greater loss of core height and a resulting expansion of the core in the diametrical direction. This increases the stress and compression in the coat ring due to higher axial and radial strains. Based on their stress and material distributions, PCTs with larger cores tend to break in a pattern that includes delamination of the coat layer. Conversely, PCTs with smaller cores tend to break around the core at higher core compression stress, suggesting different porosities in the compartments, probably in combination with a lower strength at the interface between the compartments. This hypothesis of lower strength at the interface is not proven by the data in this manuscript, but will be evaluated in future research.

The results regarding the compatibility of the recovered DCP cores show very different trends depending on core size and core compression stress. This demonstrates how the triaxial stress state during coat compression especially drastically affects the mechanical properties of core. The elastic properties of the formulations for the different compartments should be characterised in the axial and radial directions. Differences in these parameters between the compartment formulations may hint at locally acting residual stresses at the interfaces of the compartments. In the future, such data may be used to assess the propensity to pose challenges during process development and production of PCTs.

The acquired knowledge can be applied to develop press-coating process chains to yield desired, homogeneous and robust porosity and property distributions. THz-TDS proved to be superior in determining reasonable porosities than the error-prone manual preparation of the core and coat compartments. Furthermore, the non-destructive THz-TDS holds the potential to serve as a 100% online quality control method in production in the future. Further efforts will comprise the characterisation of the effect of different deformation behaviours of the coat and core formulations and determining the strength at the interfaces between the core and the coat. In future research, a model will be developed to account for elastic recovery and Poisson's ratios and calculate internal stresses that may cause fatal defects directly after compression or over storage time.

## CRediT authorship contribution statement

**Henry Brauns:** Writing – review & editing, Writing – original draft, Visualization, Validation, Methodology, Investigation, Data curation, Conceptualization. **Chi Ki Leung:** Writing – review & editing, Validation, Methodology, Investigation. **Jasper N. Ward-Berry:** Validation, Methodology, Investigation. **J. Axel Zeitler:** Writing – review & editing, Supervision, Project administration, Funding acquisition, Conceptualization. **Jan Henrik Finke:** Writing – review & editing, Writing – original draft, Supervision, Project administration, Funding acquisition, Conceptualization.

## Funding sources

RCPE is funded within the framework of COMET - Competence Centers for Excellent Technologies by BMK, BMAW, Land Steiermark, and SFG.

## Declaration of competing interest

The authors declare the following financial interests/personal relationships which may be considered as potential competing interests:

Henry Brauns reports equipment, drugs, or supplies was provided by JRS Pharma. Henry Brauns reports article publishing charges was provided by Project DEAL. Given his role as member of editorial board of the International Journal of Pharmaceutics, J. Axel Zeitler had no involvement in the peer review of this article and had no access to information regarding its peer review. Full responsibility for the editorial process for this article was delegated to another journal editor. If there are other authors, they declare that they have no known competing financial interests or personal relationships that could have appeared to influence the work reported in this paper.

## Data Availability

Data will be made available on request.

## References

[bb0005] Abdel-Hamid S., Betz G. (2011). Study of radial die-wall pressure changes during pharmaceutical powder compaction. Drug Dev. Ind. Pharm..

[bb0010] Ascani S., Berardi A., Bisharat L., Bonacucina G., Cespi M., Palmieri G.F. (2019). The influence of core tablets rheology on the mechanical properties of press-coated tablets. Eur. J. Pharm. Sci..

[bb0015] Busignies V., Mazel V., Diarra H., Tchoreloff P. (2013). Role of the elasticity of pharmaceutical materials on the interfacial mechanical strength of bilayer tablets. Int. J. Pharmaceut..

[bb0020] Cabiscol R., Finke J.H., Zetzener H., Kwade A. (2018). Characterization of Mechanical Property Distributions on Tablet Surfaces. Pharmaceutics.

[bb0025] Carstensen J.T., Toure P. (1980). Compression cycles in tableting. Powder Technol..

[bb0030] Carstensen J.T., Alcorn G.J., Hussain S.A., Zoglio M.A. (1985). Triaxial compression of “cappable” formulations. J. Pharm. Sci..

[bb0035] Chang S.-Y., Sun C.C. (2019). Insights into the effect of compaction pressure and material properties on interfacial bonding strength of bilayer tablets. Powder Technol..

[bb0040] Cocolas H.G., Lordi N.G. (1993). Axial to radial pressure transmission of tablet excipients using a novel instrumented die. Drug Dev. Ind. Pharm..

[bb0045] Diarra H., Mazel V., Busignies V., Tchoreloff P. (2015). Investigating the effect of tablet thickness and punch curvature on density distribution using finite elements method. Int. J. Pharmaceut..

[bb0050] Fell J.T., Newton J.M. (1970). Determination of tablet strength by the diametral-compression test. J. Pharm. Sci..

[bb0055] Galen S., Zavaliangos A. (2005). Strength anisotropy in cold compacted ductile and brittle powders. Acta Mater..

[bb0060] Higuchi T., Shimamoto T., Eriksen S.P., Yashiki T. (1965). Physics of tablet compression. XIV. Lateral die wall pressure during and after compression. J. Pharm. Sci..

[bb0065] Kaljević O., Djuriš J., Djurić Z., Ibrić S. (2016). Application of failure mode and effects analysis in quality by design approach for formulation of carvedilol compression coated tablets. J. Drug Delivery Sci. Technol..

[bb0070] Kim S.H., Kook J.H., Seo D.-W., Kang M.J. (2023). The effect of compression pressure on the first layer surface roughness and delamination of metformin and Evogliptin bilayer and Trilayer tablets. Pharmaceuticals (Basel).

[bb0075] Krycer I., Pope D.G., Hersey J.A. (1982). An evaluation of the techniques employed to investigate powder compaction behaviour. Int. J. Pharmaceut..

[bb0080] Latha K., Uhumwangho M.U., Sunil S.A., Srikanth M.V., Murthy K.V. (2011). Development of an optimised losartan potassium press-coated tablets for chronotherapeutic drug delivery. Trop. J. Pharm. Res..

[bb0085] Lin S.-Y., Kawashima Y. (2012). Current status and approaches to developing press-coated chronodelivery drug systems. J. Control. Release.

[bb0090] Lin S.-Y., Li M.-J., Lin K.-H. (2004). Hydrophilic excipients modulate the time lag of time-controlled disintegrating press-coated tablets. AAPS Pharm. Sci. Tech..

[bb0095] Mazel V., Busignies V., Diarra H., Tchoreloff P. (2013). On the links between elastic constants and effective elastic behavior of pharmaceutical compacts: importance of poisson’s ratio and use of bulk modulus. J. Pharm. Sci..

[bb0100] Mazel V., Yost E., Sluga K.K., Nagapudi K., Muliadi A.R. (2024). The effect of unloading and ejection conditions on the properties of pharmaceutical tablets. Int. J. Pharmaceut..

[bb0105] Meng H., Yang Y., Wu L. (2021). Strength, deformation, and acoustic emission characteristics of raw coal and briquette coal samples under a triaxial compression experiment. ACS Omega.

[bb0110] Michrafy A., Diarra H., Dodds J.A. (2009). Compaction behavior of binary mixtures. Powder Technol..

[bb0115] Nguyen T.-T., Park H.-R., Cho C.-H., Hwang K.-M., Park E.-S. (2020). Investigation of critical factors affecting mechanical characteristics of press-coated tablets using a compaction simulator. Int. J. Pharmaceut..

[bb0120] Obiorah B. (1978). Possible prediction of compression characterestics from pressure cycle plots. Int. J. Pharmaceut..

[bb0125] Pengjin Y., Shengjun M., Meifeng C., Shigui Du, Yunjin H. (2024). Real-time porosity inversion of rock based on the ultrasonic velocity and its compression-damage coupled model under triaxial compression. Sci. Rep..

[bb0130] Picart L., Mazel V., Moulin A., Tchoreloff P. (2021). Effect of the compaction parameters on the final structure and properties of a press-coated tablet (Tab-in-Tab): Experimental and numerical study of the influence of core and shell dimensions. Int. J. Pharmaceut..

[bb0135] Picart L., Mazel V., Moulin A., Bourgeaux V., Tchoreloff P. (2022). Breaking patterns of press-coated tablets during the diametral compression test: influence of the product, geometry and process parameters. Int. J. Pharmaceut..

[bb0140] Picart L., Mazel V., Moulin A., Bourgeaux V., Tchoreloff P. (2022). Controlling the lag-time and release kinetics of press-coated tablets using process parameters and tablet geometry. Int. J. Pharmaceut..

[bb0145] Picart L., Mazel V., Moulin A., Bourgeaux V., Tchoreloff P. (2022). Influence of the punch shape on the core and shell structure of press-coated tablets. Int. J. Pharmaceut..

[bb0150] Picker K.M. (2001). Time dependence of elastic recovery for characterization of tableting materials. Pharm. Dev. Technol..

[bb0155] Radojevic J., Yost E., Zavaliangos A. (2021). Evaluation of the tensile strength of compacts using square samples produced through triaxial decompression. Powder Technol..

[bb0160] Rujivipat S. (2010).

[bb0165] Sinka C. (2007). Modelling powder compaction. KONA.

[bb0170] Sugimori K.-I., Mori S., Kawashima Y. (1990). Application of a newly defined capping index in evaluation of the compressibility of pharmaceutical powders. Adv. Powder Technol..

[bb0175] Takeuchi H., Nagira S., Yamamoto H., Kawashima Y. (2004). Die wall pressure measurement for evaluation of compaction property of pharmaceutical materials. Int. J. Pharmaceut..

[bb0180] Vaithiyalingam S.R., Sayeed V.A. (2010). Critical factors in manufacturing multi-layer tablets--assessing material attributes, in-process controls, manufacturing process and product performance. Int. J. Pharmaceut..

[bb0185] Vreeman G., Sun C.C. (2022). Stress transmission coefficient is a reliable and robust parameter for quantifying powder plasticity. Powder Technol..

[bb0190] Windheuser J.J., Misra J., Eriksen S.P., Higuchi T. (1963). Physics of tablet compression. XIII. Development of die-wall pressure during compression of various materials. J. Pharm. Sci..

[bb0195] Zakowiecki D., Kukuls K., Cal K., Pelloux A., Mohylyuk V. (2025). Investigating the mechanical behaviour of viscoelastic and brittle pharmaceutical excipients during tabletting: revealing the unobvious potential of advanced compaction simulation. Pharmaceutics.

[bb0200] Zhang J., Wu C.-Y., Storey D., Byrne G. (2018). Interfacial strength of bilayer pharmaceutical tablets. Powder Technol..

